# Supplementary figures and images for: Autophagy prevents early proinflammatory responses and neutrophil recruitment during Mycobacterium tuberculosis infection without affecting pathogen burden in macrophages
Source: PLoS Biol. 2023 Jun 15;21(6):e3002159. doi: 10.1371/journal.pbio.3002159 (PMC10306192; doi:10.1371/journal.pbio.3002159)

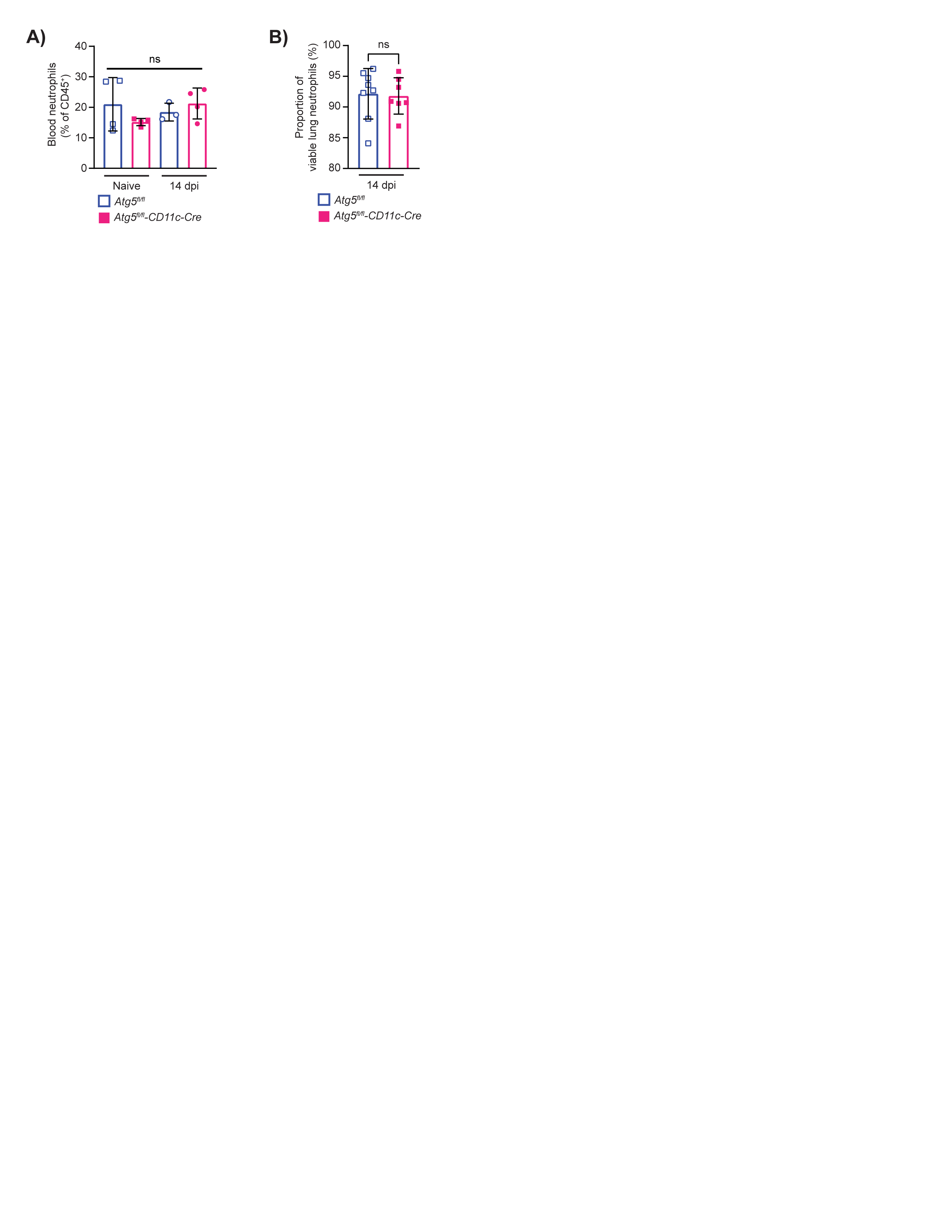

Supplement: S1 Fig — (A) The proportion of lung neutrophils (CD45+ Ly6G+ CD11b+) that are viable (Zombie-) in M. tuberculosis-infected Atg5fl/fl (n = 8) and Atg5fl/fl-CD11c-Cre (n = 7) mice at 14 dpi. (B) The proportion of CD45+ cells in the blood that are neutrophils in Atg5fl/fl (n = 4 in naïve and n = 3 in 14 dpi) and Atg5fl/fl-CD11c-Cre (n = 4) mice at 14 dpi and uninfected (naïve) mice. Statistical differences were determined by Student t test comparing the genotypes within a particular cell type or treatment group. * P < 0.05, ** P < 0.01, *** P < 0.001, **** P < 0.0001. Differences that are not statistically significant are designated as ns. Pooled data from at least 2 separate experiments are graphed where each data point is from 1 biological replicate. The individual numerical values used to generate the graphed data in S1 Fig, the statistical analyses performed to analyze these data, and the p values from these statistical tests are in S6 Data. (TIF) [file pbio.3002159.s001.tif]

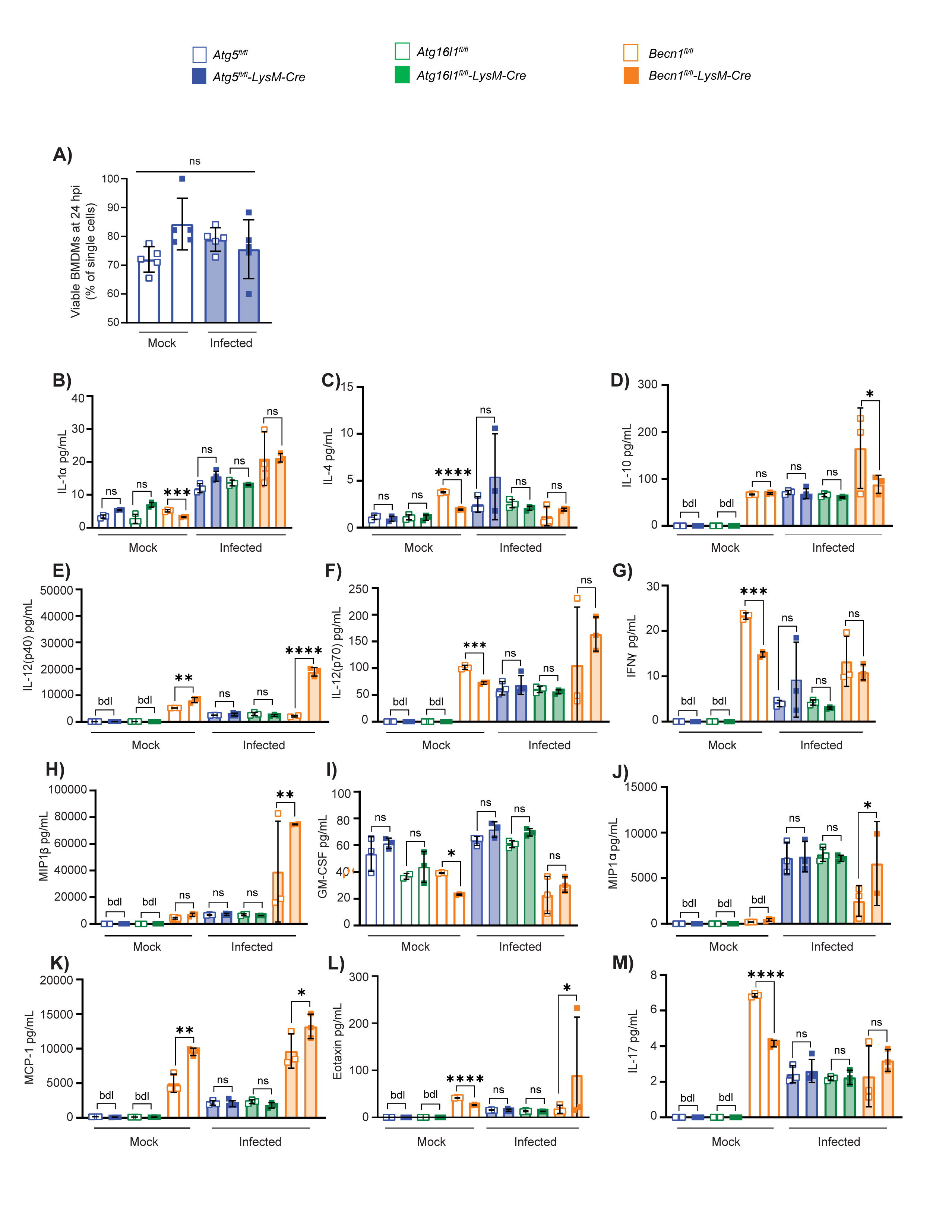

Supplement: S2 Fig — (A) The proportion of Atg5fl/fl (n = 5) and Atg5fl/fl-LysM-Cre (n = 5) BMDMs that are viable (Zombie-) at 24 hpi in mock and Mtb-GFP treated groups. Cytokine bead array data from mock treated and Mtb-GFP infected BMDMs. Atg5fl/fl, Atg5fl/fl-LysM-Cre, Atg16l1fl/fl, Atg16l1fl/fl-LysM-Cre, Becn1fl/fl, and Becn1fl/fl-LysM-Cre BMDMs were cultured for 24 hpi, and cytokine levels were measured in the spent media from mock treated or infected macrophages. BMDMs from at least 3 mice were tested in duplicate to quantify the cytokines in the bead array. All cytokine and chemokine data that are not significantly different between Atg5fl/fl-LysM-Cre and Atg5fl/fl mice are reported here. (B) IL-1α, (C) IL-4, (D) IL-10, (E) IL-12(p40), (F) IL-12(p70), (G) IFN-γ, (H) MIP1β, G (I) M-CSF, (J) MIP1α, (K) MCP-1, (L) Eotaxin, and (M) IL-17 levels at 24 hpi. Statistical differences were determined by one-way ANOVA and Šídák multiple comparison test (A) and Student t test comparing the autophagy-deficient macrophage with its floxed control within a treatment condition (B–M). * P < 0.05, ** P < 0.01, *** P < 0.001, **** P < 0.0001. Cytokine levels below detection limits are designated as dbl. Differences that are not statistically significant are designated as ns. Each data point is 1 biological replicate, and the samples were generated from at least 2 separate experiments. The individual numerical values used to generate the graphed data in S2 Fig, the statistical analyses performed to analyze these data, and the p values from these statistical tests are in S7 Data. (TIF) [file pbio.3002159.s002.tif]

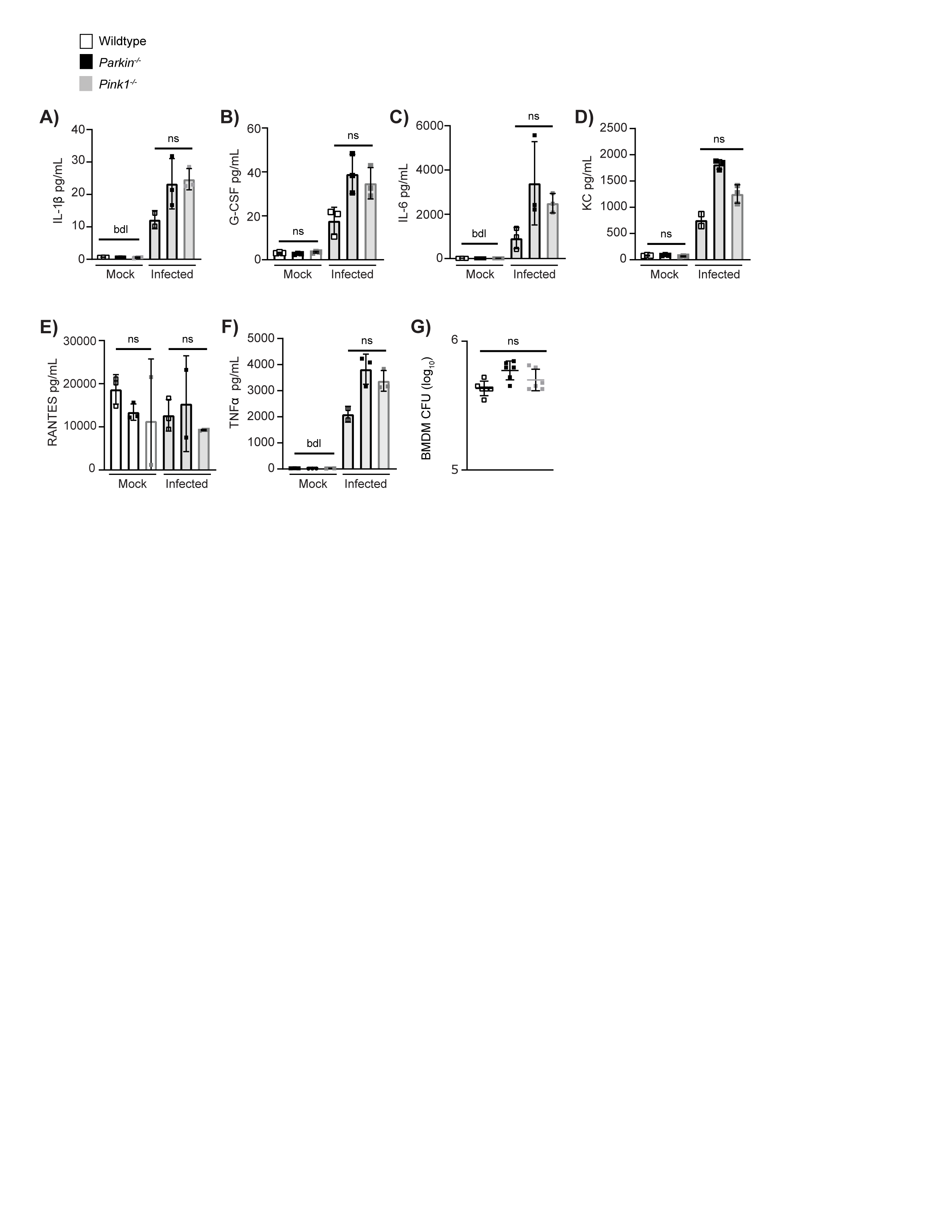

Supplement: S3 Fig — WT, Parkin-/- and Pink1-/-, BMDMs were cultured for 24 hpi and cytokine levels were measured by cytokine bead array in the media from mock treated or Mtb-GFP infected macrophages. (A) IL-1β, (B) G-CSF, (C) IL-6, (D) KC, (E) RANTES, and (F) TNF-α levels at 24 hpi (G) BMDM CFU counts from 24 hpi. BMDMs from at least 3 mice were tested in duplicate to quantify cytokines in the bead array. Each point is 1 biological replicate. Statistical differences were determined by one-way ANOVA and Šídák multiple comparison test (A–G). * P < 0.05, ** P < 0.01, *** P < 0.001, **** P < 0.0001. Cytokine levels below detection limits are designated as dbl. Statistical differences that are not significant are designated as ns. Each data point is 1 biological replicate, and the samples were generated from at least 2 separate experiments. The individual numerical values used to generate the graphed data in S3 Fig, the statistical analyses performed to analyze these data, and the p values from these statistical tests are in S8 Data. (TIF) [file pbio.3002159.s003.tif]

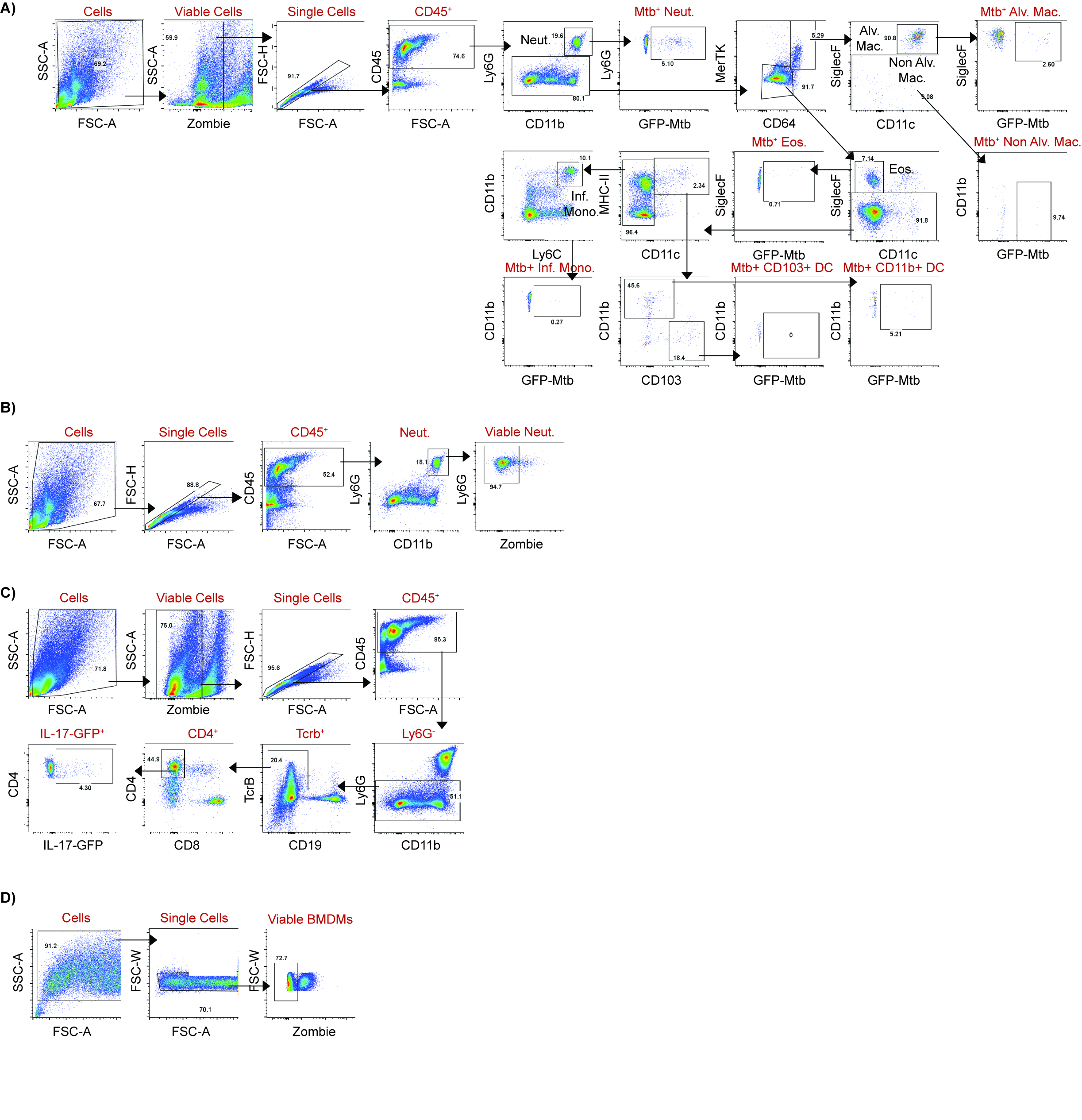

Supplement: S4 Fig — Representative flow cytometry plots depicting the gating strategy used to identify Mtb-GFP+ myeloid cells (A), viable lung neutrophils (B), and IL-17-GFP+ CD4+ T cells (C) in the lung at 14 dpi. (D) Representative flow cytometry plots showing the gating strategy to identify viable BMDMs at 24 hpi. FSC-A, forward scatter area; SSC-A, side scatter area; FSC-H, forward scatter height; FSC-W, forward scatter width, Alv. Mac., alveolar macrophages; Eos., eosinophils; DC, dendritic cell, and Inf. Mono., inflammatory monocytes. (TIF) [file pbio.3002159.s004.tif]
